# The association of vitamin D deficiency with psychiatric distress and violence behaviors in Iranian adolescents: the CASPIAN-III study

**DOI:** 10.1186/s40200-015-0191-9

**Published:** 2015-07-22

**Authors:** Asal Ataie-Jafari, Mostafa Qorbani, Ramin Heshmat, Gelayol Ardalan, Mohammad Esmaeil Motlagh, Hamid Asayesh, Seyed Masoud Arzaghi, Mohammad Hasan Tajadini, Sara Nejatinamini, Parinaz Poursafa, Roya Kelishadi

**Affiliations:** Chronic Diseases Research Center, Endocrinology and Metabolism Population Sciences Institute, Endocrinology and Metabolism Research Institute, Tehran University of Medical Sciences, Tehran, Iran; Department of Nutrition, Science and Research Branch, Islamic Azad University, Tehran, Iran; Department of Community Medicine, School of Medicine, Alborz University of Medical Sciences, Karaj, Iran; Department of Pediatrics, Child Growth and Development Research Center, Research Institute for Primordial Prevention of Non-communicable Disease, Isfahan University of Medical Sciences, Isfahan, Iran; Department of Pediatrics, Faculty of Medicine, School of Medicine, Ahvaz Jundishapur University of Medical Sciences, Ahvaz, IraN; Department of Medical Emergencies, Qom University of Medical Sciences, Qom, Iran; Elderly Health Research Center, Endocrinology and Metabolism Population Sciences Institute, Tehran University of Medical Sciences, Tehran, Iran; Department of Biotechnology, School of Pharmacy, Isfahan University of Medical Sciences, Isfahan, Iran

**Keywords:** Mental health, Violence behaviors, Anger, Anxiety, Depression, Vitamin D, Adolescents

## Abstract

**Background:**

Subtle effects of vitamin D deficiency on behavior have been suggested. We investigated the association of vitamin D status with mental health and violence behaviors in a sample of Iranian adolescents.

**Methods:**

This nationwide study was conducted in 2009–2010 in 1095 Iranian school students with mean age 14.7 ± 2.6 years. Items were adapted from the Global School-based Student Health Survey (GSHS). Psychiatric distress was considered as the self-reported anger, anxiety, poor quality sleep, confusion, sadness/depression, worry, and violence-related behaviors (physical fight, having bully, or getting bullied).

**Results:**

Forty percent had serum 25(OH)D values below 10 ng/mL (vitamin D deficient), and 39 % had levels 10-30 ng/mL (vitamin D insufficient). The prevalence of self-reported anger, anxiety, poor quality sleep, sadness/depression, and worry was significantly lower (*P* < 0.05) in vitamin D sufficient participants compared with their other counterparts. The odds of reporting anger, anxiety, poor quality sleep, and worry, increased approximately 1.5 to 1.8 times in vitamin D insufficient compared with normal children and adolescents (*P* < 0.05). Risk estimates indicated that vitamin D insufficient and deficient subjects had higher odds of reporting worry compared to normal vitamin D group [OR = 2.417 (95 % CI: 1.483-3.940) for vitamin D insufficient students, and OR = 2.209 (95 % CI: 1.351-3.611) for vitamin D deficient students] (P-trend = 0.001). Violence behaviors did not show any association with vitamin D status (*P* > 0.05).

**Conclusion:**

Some psychiatric distress such as anger, anxiety, poor quality sleep, depression, and worry are associated with hypovitaminosis D in adolescents. The clinical significance of the current findings should be determined in future longitudinal studies.

## Introduction

Mental disorders are one of the most common health problems worldwide. About half of all mental disorders begin before the age of 14 [1]. The World Health Organization (WHO) predicts that by the year 2020, childhood neuropsychiatric disorders will rise to become one of the five most common causes of mortality, morbidity, and disability among children [2].

Studies in various countries including Egypt, Nigeria, India, Indonesia, Thailand, and Sri Lanka [3] have shown that rates of child and adolescent mental disorders are comparable to rates reported in developed countries [4]. Iran, as a developing country, is undergoing significant social, cultural, and economic changes, which affect its populations' mental health status.

Few studies have been conducted to evaluate mental health status in children and adolescents in Iran. Two surveys with similar methodology in Tehran have shown the prevalence of overall psychological disorders to be 17.9 % in 7-12-year old children [5] and 14.2 % in 12–17 year-old adolescents [6]. A systematic review of studies conducted among high school students in Iran showed that the prevalence rates of mental disorders were reported in a wide range from 4.34 % to 16.6 % in studies using diagnostic instruments to 34.4 % in studies using screening instruments [7].

Vitamin D is a hormone with key functions more than calcium homeostasis and maintaining bone health. Vitamin D receptors are present in a wide variety of cells, including neurons and glial cells. Genes encoding the enzymes involved in the metabolism of vitamin D are also expressed in the brain [8]. Vitamin D promotes neurogenesis and regulates the synthesis of neurotrophic factors, which support differentiation of neurons and survival [9, 10].

Most epidemiological evidence supports a link between vitamin D deficiency and mental disorders in adults. A recent British study showed that low levels of vitamin D_3_ are associated with increased risk of common mental disorders in mid-adulthood [11]. Low serum 25-hydroxyvitamin D [25(OH)D] has been also associated with increased odds of cognitive impairment [12–14] and depressive symptoms in adults [15–17], but some inconsistencies exist between different studies [18–21].

The purpose of this study was to investigate the association of vitamin D deficiency with psychiatric distress and violence behaviors in a nationally representative sample of Iranian children and adolescents.

## Materials and methods

The data used in this study were obtained as part of the third national survey of school student high risk behaviors (2009–2010) in Iran entitled “**C**hildhood and **A**dolescence **S**urveillance and **P**revent**I**on of **A**dult **N**on-communicable Disease” (CASPIAN) study.

Details on the survey design and methods have been explained previously [22]. In brief, 5570 school students aged 10–18 years, living in urban and rural areas in 27 provinces of Iran were selected via multistage-random cluster sampling method. Eligible schools in this survey were stratified according to the information bank of the Ministry of Health and Medical Education and then, they were selected randomly. In selected schools, the students were selected via random sampling method. Ethical committees and relevant national organizations approved the study. Oral assent was obtained from students, and written informed consent from their parents. A team of trained health care professionals checked the performance of the personnel, monitored and calibrated equipment according to standard protocols.

As described before, the current study was performed in a sub-sample randomly selected among participants in the main study. It was approved by the Research Ethics Committee of Isfahan University of Medical Sciences, Isfahan, Iran. The sample size of the current study was calculated by assuming an alpha error of 5 % and a power of 80 % and design effect of 1.25, while adding 25 % to the estimated sample size. The final sample size was calculated as 1000, and for possible missing data, we increased it by 10 % and studied 1100 samples [23].

### Clinical and laboratory measurements

Weight was measured on calibrated scales to the nearest 0.1 kg while subjects wearing light clothing, and height were measured without shoes to the nearest 0.1 cm. Body mass index (BMI) was calculated as weight (kg) divided by height squared (m^2^). Waist circumference was measured using a non-elastic tape at a point midway between the lower border of the rib cage and the iliac crest at the end of normal expiration to the nearest 0.1 cm.

Blood samples were collected in the morning after 10–12 h overnight fasting. Serum concentration of 25(OH)D was analyzed quantitatively by direct competitive immunoassay chemiluminescene method using LIASON® 25 OH vitamin D assay TOTAL (DiaSorin, Inc.), with a coefficient of variation (CV) of 9.8 %. Serum 25(OH)D level of less than 10 ng/mL was considered as vitamin D deficiency and levels between 10-30 ng/mL as vitamin D insufficiency [24].

Demographic information was completed by obtaining data for all officially enrolled students in the sampled classes from the school record. Demographic and anthropometric information was collected based on the Persian version of main questionnaire of the World Health Organization- Global School-based student Health Survey (WHO-GSHS).

Parental level of education, possessing a family private car and type of home, physical activity, sedentary lifestyle, birth weight, breast feeding duration, type of milk and type of complementary feeding in childhood were assessed through two sets of questionnaires for students and parents which were filled in under the supervision of the trained health professionals.

Having personal home, personal car, and personal computer was used as some components of socio-economic status. Breastfeeding duration was defined as the whole month that participants were breast fed (exclusively or in combination with other foods). Complementary feeding was asked as home-made foods or commercial baby food. Sedentary behavior was assessed by watching TV and working computer.

### Psychiatric distress and violence variables

In this study, we used part of Global School-based Student Health Survey (GSHS) questionnaire from WHO for information regarding psychiatric distress and violence behaviors. The validity of questionnaire has been evaluated in 120 urban and rural students in one of the regions around Tehran (the Cronbach’s reliability coefficient >0.7) [25].

Psychiatric distress included one of the angriness, anxiety, insomnia, confusion, sadness/depression, and worry, which were reported on a Likert scale questionnaire by students. In addition, some questions about violence and students’ perceived general health status were asked. All factors were categorized as binary variables. The questions, codes and categorization have been shown in Appendix 1.

### Statistical analysis

Findings on continuous variables are presented as means (SD)/median (interquartile range), and categorical data as percentages. Association between qualitative variables was assessed by using Pearson Chi-square test. The normality of continuous variables was assessed by Kolmogorov-Smirnov test, and due to lack of normality of serum concentrations of 25(OH)D, the Mann–Whitney *U* test was used to compare the median values of serum25(OH)D across psychiatric distress categories.

Logistic regression analysis was applied to determine the association of 25(OH) D status (as continuous and categorical variable) with psychiatric distress in three models: Model I, crude model (without adjustment); Model II, adjustment for age, sex, and living area; and Model III, additionally adjustment for other potential confounders, including sleeping hours, socio-economic status, physical activity, breast feeding, type of complementary feeding, BMI, and type of milk used in infancy. The results of logistic regression are shown as odds ratios (OR) and 95 % confidence interval (CI). Data were analyzed by SPSS statistical software (version 16.0; SPSS Inc., Chicago, IL, USA); the significance level was set at *P* < 0.05.

## Results

Among the 1095 children and adolescents included in the analysis (mean age 14.7 ± 2.6 years; mean BMI 19.3 ± 4.2 kg/m^2^), the median serum 25(OH)D was 13.0 ng/mL(interquartile range 6.8-27.4 ng/mL). A total of 40 % were vitamin D deficient and 39 % were vitamin D insufficient.

Baseline characteristics of subjects according to vitamin D status (normal, deficient or insufficient) and by sex are shown in Table 1. Participants with different status of vitamin D were comparable in case of anthropometric measurements, duration of breast- feeding, type of milk used in infancy, birth weight, physical activity or sedentary behavior, and socio-economic status. The only significant difference was documented for the type of complementary feeding in childhood among normal, vitamin D deficient and vitamin D insufficient girls (*P*-trend = 0.022) (Table 1).

**Table 1:** General characteristics of participants according to vitamin D status categories: the CASPIAN-III Study

Variables	Vitamin D status (boys) *N* = 568				*P*-value^a^	Vitamin D status (girls) *N* = 527				*P*-value^a^	Vitamin D status (total) *N* = 1095				*P*-value^a^
	Deficient	Insufficient	Normal	Total		Deficient	Insufficient	Normal	Total		Deficient	Insufficient	Normal	Total	
Age (y)	14.7 ± 2.5	14.6 ± 2.3	14.7 ± 2.7	14.6 ± 2.5	0.870	14.9 ± 2.7	14.9 ± 2.7	14.8 ± 2.8	14.9 ± 2.7	0.950	14.8 ± 2.6	14.7 ± 2.6	14.7 ± 2.7	14.7 ± 2.6	0.803
Weight (kg)	46.9 ± 22.6	45.6 ± 13.9	45.6 ± 13.4	46.2 ± 17.9	0.665	47.8 ± 16.9	47.9 ± 18.8	48.7 ± 18.9	48.0 ± 18.0	0.915	47.4 ± 20.1	46.7 ± 16.6	47.0 ± 16.1	47.0 ± 17.9	0.726
Height (Cm)	150.2 ± 17.0	150.6 ± 11.9	151.7 ± 12.3	150.7 ± 14.3	0.605	155.6 ± 17.4	155.4 ± 17.2	155.8 ± 15.2	155.6 ± 16.9	0.980	152.7 ± 17.4	153.0 ± 15.0	153.5 ± 13.8	153.0 ± 15.8	0.514
Waist circumference (Cm)	66.2 ± 9.4	66.0 ± 11.2	67.4 ± 10.2	66.4 ± 10.3	0.443	68.4 ± 13.3	70.4 ± 15.6	69.2 ± 11.4	69.4 ± 13.9	0.351	67.2 ± 11.4	68.2 ± 13.7	68.2 ± 10.8	67.8 ± 12.2	0.260
BMI (kg/m^2^)	19.4 ± 4.0	19.8 ± 4.6	19.4 ± 3.8	19.5 ± 4.2	0.618	18.9 ± 4.0	18.9 ± 4.3	19.2 ± 3.7	18.9 ± 4.1	0.782	19.2 ± 4.0	19.3 ± 4.5	19.3 ± 3.7	19.3 ± 4.2	0.867
Breast feeding duration (months)	16.70 ± 8.0	16.4 ± 8.0	15.6 ± 8.2	16.3 ± 8.1	0.482	15.3 ± 8.1	16.5 ± 7.5	16.1 ± 8.8	15.9 ± 8.0	0.324	16.03 ± 8.1	16.43 ± 7.7	15.82 ± 8.5	16.1 ± 8.0	0.636
Type of milk used in infancy															
Breast fed	83.5	85.8	75.4	82.5		77.3	85.5	79.0	81.0		80.6	85.7	77.0	81.8	
Formula	4.5	4.4	8.2	5.3	0.178	3.9	2.3	5.0	3.5	0.245	4.2	3.3	6.8	4.4	0.053
Mixed	12.1	9.8	16.4	12.2		18.7	12.1	16.0	15.5		15.2	11.0	16.2	13.8	
Type of complementary feeding (%)															
Always home-made food	55.8	60.9	60.3	58.7		63.2	60.6	58.0	61.1		59.3	60.6	59.3	59.8	
Always commercial baby food	7.1	4.8	2.4	5.2	0.378	3.5	1.4	7.0	3.3	0.022	5.4	3.1	4.4	4.3	0.086
Usually home-made foods^b^	29.0	29.0	32.5	29.8		24.9	33.8	32.0	30.0		27.1	31.6	32.3	30.0	
Usually commercial baby food^c^	8.0	5.3	4.8	6.3		8.5	4.2	3.0	5.6		8.2	4.7	4.0	6.0	
Birth weight (g)															
<2500	15.1	17.9	16.1	16.4		16.3	11.1	16.2	14.1		15.6	14.4	16.1	15.3	
2500-4000	74.9	76.4	76.6	75.8	0.525	75.9	77.4	72.7	75.9	0.399	75.4	77.0	74.9	75.9	0.973
>4000	10.0	5.6	7.3	7.8		7.9	11.5	11.1	10.0		9.0	8.7	9.0	8.9	
Watching TV (%)															
<2 h	49.1	53.8	53.5	51.9	0.562	50.7	51.0	45.5	49.8	0.633	49.9	52.5	50.0	50.9	0.708
>2 h	50.9	46.2	46.5	48.1		49.3	49.0	54.5	50.2		50.1	47.5	50.0	50.1	
Working with computer (%)															
<2 h	91.0	94.6	96.0	93.5	0.137	88.6	86.7	84.7	87.1	0.634	89.9	90.7	91.1	89.9	0.864
>2 h	9.0	5.4	4.0	6.5		11.4	13.3	15.3	12.9		10.1	9.3	8.9	9.6	
Father’s education (%)															
Illiterate	11.0	13.9	15.7	13.2		17.1	16.4	9.9	15.4		13.8	15.1	13.2	14.2	
Elementary to high school	83.3	77.1	81.1	80.5	0.171	72.4	73.4	74.3	73.2	0.330	78.2	75.2	78.1	77.0	0.846
College	5.7	9.0	3.1	6.3		10.6	10.3	15.8	11.5		8.0	9.6	8.8	8.8	
Mother’s education (%)															
None	20.0	16.5	17.8	18.2		23.8	24.3	15.8	22.5		21.8	20.5	17.0	20.2	
Elementary to high school	77.4	76.7	79.8	77.7	0.148	71.3	71.1	73.3	71.6	0.104	74.5	73.9	77.0	74.8	0.368
College	2.6	6.8	2.3	4.1		5.0	4.6	10.9	6.0		3.7	5.6	6.1	5.0	
Socio-economic status (%)															
Personal home	85.8	88.2	88.2	87.2	0.699	84.4	81.9	81.8	82.9	0.761	85.1	85.0	85.4	85.2	0.992
Rented home	14.2	11.8	11.8	12.8		15.6	18.1	18.2	17.1		14.9	15.0	14.6	14.8	
Personal car (%)															
Yes	49.1	50.2	47.2	49.1	0.869	47.8	44.7	55.0	47.9	0.231	48.5	47.5	50.7	48.6	0.744
No	50.9	49.8	52.8	50.9		52.2	55.3	45.0	52.1		51.5	52.5	49.3	51.4	
Personal computer (%)															
Yes	38.9	44.7	35.7	40.3	0.219	41.3	39.0	49.5	41.9	0.203	40.0	41.9	41.7	41.1	0.829
No	61.1	55.3	64.3	59.7		58.7	61.0	50.5	58.1		60.0	58.1	58.3	58.9	
Sleeping duration (hours)	8.9 ± 2.2	8.9 ± 1.9	9.2 ± 2.3	9.0 ± 2.1	0.358	9.1 ± 2.2	9.2 ± 2.1	8.7 ± 2.2	9.0 ± 2.2	0.137	9.0 ± 2.2	9.1 ± 2.0	9.0 ± 2.3	9.0 ± 2.1	0.908
Physical activity (hour/week)	2.9 ± 1.5	2.9 ± 1.6	2.9 ± 1.4	2.9 ± 1.5	0.932	3.6 ± 2.4	3.6 ± 1.4	3.6 ± 1.6	3.6 ± 1.9	0.936	3.3 ± 2.0	3.2 ± 1.5	3.2 ± 1.5	3.2 ± 1.7	0.866
Living area (%)															
Urban	64.5	67.8	68.2	66.5	0.691	65.5	70.6	65.7	67.7	0.474	65.0	69.1	67.1	67.0	0.440
Rural	35.5	32.2	31.8	33.5		34.5	29.4	34.3	32.3		35.0	30.9	32.9	33.0	
Type of dairy used by students															
Low-fat	81.9	84.3	82.9	83.0	0.795	87.4	83.3	80.2	84.3	0.249	84.4	83.8	81.7	83.6	0.675
High-fat	18.1	15.7	17.1	17.0		12.6	16.7	19.8	15.7		15.6	16.2	18.3	16.4	

^a^-Comparisons based on *χ*2 test or independent samples *t* test, as appropriate

^b^-It means using home-made foods, but sometimes using commercial baby foods

^c^-It means using commercial baby foods, but sometimes using home-made foods

Table 2 represents the prevalence of psychosocial disorders according to the vitamin D status (normal, deficient or insufficient) in boys and girls. The prevalence of self-reported angriness, anxiety, poor quality sleep, sadness/depression, and worry was significantly lower in vitamin D sufficient subjects compared with their other counterparts (P <0.05).

**Table 2:** Prevalence of psychiatric distress according to vitamin D status: the CASPIAN III study

Variables	Vitamin D status (boys) *N* = 568				*P*-value^b^	Vitamin D status (girls) *N* = 527				*P*-value^b^	Vitamin D status (total)				*P*-value^b^
	Deficient^a^	Insufficient^a^	Normal^a^	Total		Deficient^a^	Insufficient^a^	Normal^a^	Total		Deficient^a^	Insufficient^a^	Normal^a^	Total	
Angriness (Yes) %	67.5	74.0	57.4	67.6	0.006	65.2	65.6	53.9	63.2	0.097	66.4	69.6	55.8	65.4	0.002
Anxiety (Yes) %	69.7	71.6	62.8	68.8	0.219	60.9	62.4	43.1	58.1	0.003	65.5	67.0	54.1	63.7	0.003
poor quality sleep(Yes) %	48.9	43.8	35.7	44.0	0.052	46.9	49.1	40.2	46.5	0.329	47.9	46.6	37.7	45.3	0.031
Confusion (Yes) %	35.1	30.8	34.1	33.3	0.618	33.3	36.7	25.5	33.2	0.140	34.2	33.7	30.3	33.2	0.565
Sadness /Depression (Yes) %	32.9	35.2	18.7	30.2	0.021	29.9	25.0	12.5	24.3	0.021	31.4	29.5	16.0	27.2	0.001
Worry (Yes) %	31.4	23.5	18.2	25.7	0.056	27.3	25.3	20.0	25.0	0.493	29.5	24.7	19.0	25.5	0.042
Physical fight (Yes) %	46.1	45.2	39.8	44.3	0.501	58.5	58.4	59.6	58.7	0.979	52.0	51.8	48.5	51.2	0.658
Getting bullied (Yes) %	35.8	28.0	32.0	32.1	0.220	35.4	38.5	39.6	37.5	0.721	35.6	33.5	35.4	34.7	0.786
Bully (Yes) %	19.7	21.7	25.0	21.6	0.512	31.9	35.1	34.7	33.7	0.769	25.5	28.4	29.3	27.4	0.488
General health (bad) %	25.5	25.0	26.1	25.4	0.977	26.4	23.9	23.0	24.7	0.770	25.9	24.4	24.7	25.0	0.873

^a^-Normal: serum 25(OH)D > 30 ng/mL; Vitamin D insufficient: 10 < 25(OH)D < 30 ng/mL; vitamin D deficient: 25(OH)D < 10 ng/mL

^b^-Comparisons based on *χ*2 test

Table 3 shows that median levels of serum 25(OH) D were not significantly different in boys who experienced angriness during the prior 6 months compared with other boys (*P* > 0.05). However, median levels of vitamin D was significantly lower in boys who reported to have poor quality sleep, sadness/depression, or worry compared with those who did not (*P* < 0.05). In girls, the serum levels of 25(OH)D was lower in those who reported angriness or anxiety during the previous 6 months, or sadness/depression or worry during the previous 12 months compared with those who did not report them (*P* < 0.05). In total, participants who reported angriness, anxiety, poor quality sleep, sadness/depression, or worry, had lower levels of serum 25(OH)D than those who did not report such disorders (*P* < 0.05). These differences in serum 25 (OH) D levels were highly significant in reporting worry (10.1 ng/mL [interquartile range: 4.2 - 19.7] in individuals who reported worry, compared with 15.0 ng/mL [interquartile range: 7.7 - 29.2] in those without worry) (*P* < 0.001).

**Table 3:** Serum 25(OH)D concentrations (ng/mL) according to various psychiatric distress: the CASPIAN III study^a^

Variables		Boys, n = 568	*P*-value	Girls, n = 527	*P*-value	Total	*P*-value^b^
Angriness	No	12.3 (6.3, 36.0)	0.323	14.7 (7.5, 29.7)	0.042	13.7 (6.7, 31.8)	0.033
	Yes	12.8 (6.7, 25.7)		11.7 (7.2, 24.5)		12.4 (6.9, 25.3)	
Anxiety	No	14.0 (6.3, 31.9)	0.308	15.5 (7.7, 31.2)	0.004	14.6 (7.3, 31.3)	0.007
	Yes	12.4 (6.6, 26.9)		11.7 (6.6, 23.6)		12.2 (6.6, 25.2)	
Poor quality sleep	No	14.0 (6.8, 30.7)	0.020	13.4 (7.4, 28.3)	0.329	13.6 (6.9, 29.5)	0.016
	Yes	11.2 (6.1, 24.1)		13.2 (7.2, 24.6)		11.6 (6.6, 24.5)	
Confusion	No	12.6 (6.7, 28.2)	0.694	13.6 (7.4, 28.0)	0.258	13.0 (7.1, 28.0)	0.298
	Yes	12.9 (6.2, 28.8)		13.0 (7.1, 24.0)		12.9 (6.6, 25.6)	
Sadness/ Depression	No	13.5 (6.9, 31.5)	0.017	15.4 (8.1, 29.3)	0.001	14.7 (7.5, 30.4)	<0.001
	Yes	10.4 (5.5, 20.0)		10.1 (5.0, 18.3)		10.2 (5.2, 18.7)	
Worry	No	14.2 (7.2, 29.9)	<0.001	16.0 (8.1, 28.3)	<0.001	15.0 (7.7, 29.2)	<0.001
	Yes	7.5 (4.0, 19.8)		10.2 (4.8, 19.6)		10.1 (4.2, 19.7)	
Physical fight	No	13.4 (6.4, 29.5)	0.526	13.1 (7.4, 24.6)	0.551	13.2 (6.6, 27.6)	0.926
	Yes	11.9 (6.7, 26.1)		13.3 (7.4, 27.7)		12.5 (7.1, 26.9)	
Getting bullied	No	13.2 (6.8, 28.7)	0.433	13.6 (7.4, 27.0)	0.892	13.4 (7.0, 27.6)	0.615
	Yes	11.6 (6.3, 27.4)		13.1 (7.2, 25.8)		12.5 (6.4, 26.5)	
Bully	No	12.3 (6.4, 28.2)	0.289	13.0 (7.1, 27.2)	0.387	12.7 (6.7, 27.6)	0.171
	Yes	14.9 (6.8, 32.1)		16.3 (7.5, 26.0)		15.5(7.4, 27.3)	
General health	Good	12.3 (6.6, 26.8)	0.608	13.7 (7.5, 27.3)	0.268	13.3 (6.9, 27.1)	0.695
	Bad	13.2 (6.6, 27.9)		11.0 (6.8, 24.0)		11.7 (6.6, 25.9)	

^a^Data are median (interquartile range)

^b^Man-Whitney*U*test

Table 4 presents the association parameters (OR and 95 % CI) of vitamin D status with psychiatric distress and violence from logistic regression models. In model I, the odds of angriness increased 1.56 times in vitamin D insufficient individuals compared with their other counterparts. In vitamin D deficient subjects, the odds ratio was 1.80 times higher than vitamin D sufficient students (*P*-trend = 0.026). By the inclusion of sex, age, and living area in model II, and also by additional adjustment for sleeping hours, socio-economic status, physical activity, breast feeding, type of milk used in infancy, complementary feeding and BMI in model III, the associations were approximately the same as the crude model (*P*-trend = 0.020 and 0.015; respectively).

**Table 4:** Odds ratios (95 % CI) for psychiatric distress according to vitamin D status: the CASPIAN-III study

	Vitamin D status			*P*-trend^b^
	Normal^a^	Insufficient^a^	Deficient^a^	
Angriness				
Model I^c^	1	1.565 (1.129, 2.170)	1.806 (1.297, 2.517)	0.026
Model II^d^	1	1.577 (1.134, 2.192)	1.868 (1.336, 2.612)	0.020
Model III^e^	1	1.759 (1.185, 2.612)	2.026 (1.365, 3.008)	0.015
Anxiety				
Model I	1	1.612 (1.164, 2.231)	1.720 (1.239, 2.388)	0.013
Model II	1	1.655 (1.187, 2.308)	1.815 (1.297, 2.540)	0.009
Model III	1	1.728 (1.162, 2.570)	1.756 (1.185, 2.603)	0.015
poor quality sleep				
Model I	1	1.525 (1.101, 2.111)	1.445 (1.042, 2.004)	0.019
Model II	1	1.526 (1.102, 2.114)	1.426 (1.027, 1.979)	0.021
Model III	1	1.348 (0.917, 1.982)	1.472 (1.004, 2.157)	0.258
Confusion				
Model I	1	1.198 (0.850, 1.688)	1.170 (0.829, 1.652)	0.341
Model II	1	1.208 (0.856, 1.704)	1.170 (0.828, 1.654)	0.361
Model III	1	1.053 (0.700, 1.585)	1.012 (0.674, 1.520)	0.849
Sadness/Depression				
Model I	1	2.417 (1.483, 3.940)	2.209 (1.351, 3.611)	0.001
Model II	1	2.458 (1.504, 4.018)	2.308 (1.407, 3.785)	0.001
Model III	1	2.355 (1.325, 4.187)	2.405 (1.356, 4.267)	0.009
Worry				
Model I	1	1.786 (1.126, 2.833)	1.400 (0.873, 2.245)	0.012
Model II	1	1.808 (1.136, 2.878)	1.381 (0.858, 2.224)	0.010
Model III	1	1.484 (0.860, 2.559)	1.246 (0.718, 2.163)	0.149
Physical fight				
Model I	1	1.151 (0.834, 1.587)	1.142 (0.827, 1.577)	0.440
Model II	1	1.135 (0.820, 1.571)	1.101 (0.794, 1.527)	0.516
Model III	1	1.282 (0.871, 1.887)	1.358 (0.925, 1.992)	0.312
Getting bullied				
Model I	1	1.011 (0.724, 1.413)	0.920 (0.656, 1.290)	0.839
Model II	1	1.015 (0.724, 1.423)	0.896 (0.637, 1.262)	0.919
Model III	1	1.021 (0.680, 1.531)	0.920 (0.614, 1.378)	0.949
Bully				
Model I	1	0.826 (0.578, 1.181)	0.961 (0.673, 1.371)	0.256
Model II	1	0.812 (0.565, 1.166)	0.922 (0.643, 1.323)	0.185
Model III	1	0.781 (0.512, 1.189)	0.717 (0.471, 1.091)	0.286
General health				
Model I	1	0.936 (0.640, 1.368)	1.014 (0.691, 1.487)	0.676
Model II	1	0.934 (0.638, 1.368)	1.018 (0.693, 1.496)	0.726
Model III	1	1.003 (0.638, 1.577)	1.075 (0.687, 1.684)	0.973

^a^-Normal: serum 25(OH)D > 30 ng/mL; Vitamin D insufficient: 10 < 25(OH)D < 30 ng/mL; vitamin D deficient: 25(OH)D < 10 ng/mL

^b^-*P*-trends resulted from logistic regression

^c^-Without adjustment (crude models)

^d^-Adjusted for age, sex, and living area

^e^-Additionally adjusted for other characteristics including sleeping hours, socio-economic status, physical activity, breast feeding, complementary feeding, BMI, type of milk

Children and adolescents with vitamin D insufficiency and vitamin D deficiency were 1.61 and 1.81 times more likely to report anxiety disorders compared with their other counterparts in model I (*P*-trend = 0.013). These association were stronger in model II (*P* = 0.009), but the same as the crude model in model III (*P-*trend = 0.015). Similar associations were observed in reporting anxiety, poor quality sleep, and worry, for which subjects with vitamin D insufficiency and vitamin D deficiency were approximately 1.5 to 1.8 times more likely to report such disorders in comparison to those with normal vitamin D levels (*P* < 0.05). The strongest association was observed in case of reporting sadness/depression, the odds of which increased approximately 2.2 to 2.5 time in vitamin D insufficient and deficient compared with their vitamin D sufficient counterparts (*P* < 0.01). Other parameters, as the self-reported general health status and violence behaviors did not show any association with vitamin D status (*P* > 0.05) (Table 4).

According to the logistic regression methods, for every 1 ng/mL increase of serum 25(OH)D levels, the odds of angriness and anxiety decreased by 1-2 % (*P* < 0.001), and the likelihood of poor quality sleep, sadness/depression, and worry had the same results (*P* < 0.05) (Table 5).

**Table 5:** Association of 25(OH)D concentrations with psychiatric distress: the CASPIAN III study (*N* = 1095)

	25(OH)D concentrations (ng/mL)					
	Model I^a^	*P* value	Model II^b^	*P* value	Model III^c^	*P* value
	Odds ratio (95 % CI)		Odds ratio (95 % CI)		Odds ratio (95 % CI)	
Angriness	0.985 (0.978, 0.992)	<0.001	0.984 (0.977, 0.992)	<0.001	0.981 (0.972, 0.990)	<0.001
Anxiety	0.983 (0.976, 0.991)	<0.001	0.982 (0.974, 0.989)	<0.001	0.981 (0.973, 0.990)	<0.001
Poor quality sleep	0.988 (0.981, 0.996)	0.003	0.988 (0.981, 0.996)	0.002	0.992 (0.983, 1.001)	0.066
Confusion	0.996 (0.988, 1.004)	0.304	0.996 (0.988, 1.004)	0.285	0.999 (0.990, 1.008)	0.860
Sadness/Depression	0.989 (0.979, 0.999)	0.037	0.989 (0.979, 0.999)	0.031	0.990 (0.978, 1.001)	0.083
Worry	0.982 (0.970, 0.993)	0.001	0.982 (0.971, 0.994)	0.002	0.986 (0.973, 0.999)	0.033
Physical fight	0.997 (0.990, 1.005)	0.499	0.998 (0.991, 1.006)	0.632	0.996 (0.987, 1.005)	0.357
Getting bullied	0.998 (0.991, 1.006)	0.664	0.999 (0.991, 1.007)	0.751	0.999 (0.989, 1.008)	0.760
Bully	1.004 (0.996, 1.012)	0.317	1.005 (0.997, 1.013)	0.243	1.004 (0.995, 1.014)	0.365
General health	0.997 (0.989, 1.006)	0.559	0.998 (0.989, 1.006)	0.611	0.997 (0.987, 1.007)	0.602

^a^-Without adjustment (crude model)

^b^-Adjusted for age, sex, and living area

^c^-Additionally adjusted for other characteristics including sleeping hours, socio-economic status, physical activity, breast feeding, type of complementary feeding, BMI, type of milk used in infancy

## Discussion

This nationwide study, which to the best of our knowledge is the first of its kind in the pediatric age group, investigated the psychiatric distress, violence, and general health in relation to vitamin D status in a nationally -representative sample of Iranian children and adolescents. The study found significant associations between vitamin D deficiency and self-reported psychiatric distress as angriness, anxiety, poor quality sleep, sadness/depression, and worry. However, no significant association existed between vitamin D status and violence behaviors.

Anxiety, depression, mood disorders, and behavioral and cognitive disorders are among the most prevalent mental health problems of children and adolescents [26]. Different methods and tools could be used for screening and diagnosis of psychiatric distress. In this study, the questionnaire of GSHS was used to assess the self-reported mental health status, violence issues, and general health of students. The purpose of the GSHS is to help countries measure and assess behavioral risk factors and protective factors in 10 key areas that contribute to morbidity and mortality among children and adults.

The current study revealed significant associations between vitamin D deficiency and self-reported psychiatric distress such as angriness, anxiety, poor quality sleep, sadness/depression, and worry. To our knowledge, there is no similar study in this age group to compare our results. However, most studies in adults have documented that better vitamin D status is associated with better cognitive function and mental health [12, 27–32], but not in others [18–21]. One study found an inverse association between dietary vitamin D and depression [33], and a number of clinical trials have shown positive effects of vitamin D on mood and depression [31], but no effect of an annual high dose of vitamin D was observed on depressive symptoms in older women [34]. Pan et al. also reported no significant correlation between vitamin D status and depression in Chinese adults [18].

It is very difficult to find whether the differences between these studies are due to physiological differences or resulting from methodological aspects as study population, method of assessing psychiatric distress, timing of the blood collection, method of vitamin D assessment, and covariates considered in regression models.

The current study could adjust the associations for a range of variables including physical activity-related variables, sleeping duration, socio-economic status, breast feeding, type of milk used in infancy, type of complementary feeding, BMI, and waist circumference. In the only prospective study, which examined the association of 25(OH)D_3_ with depressive symptoms in children, the association only emerged with symptoms measured 3 years after exposure assessment, and was not present when symptoms were assessed just 1 year after exposure assessment. The causality was not confirmed, and the association was partly explained by factors other than 25(OH)D_3_ (such as outdoor physical activity) but that were associated with it and accumulated over time [35].

The mechanism through which vitamin D plays a role in mental health is not fully understood. Vitamin D is a neuro-steroid hormone which regulates the metabolism of neurotransmitters in the central nervous system [36, 37]. The function of monoamine neurotransmitters such as serotonin and norepinephrine have been known on pathophysiology of depression and mood disorders [38]. In addition, serotonin regulates stress, anger, depression, aggression, appetite, and behavior. As a result, the association between vitamin D and psychiatric distress might be mediated by serotonin levels. Some other mechanisms have been also proposed for the potentially influence of vitamin D on brain function. Vitamin D receptors (VDRs), 25(OH)D 1-α-hydroxylase, and the cytochrome P-450 that catalyzes the hydroxylation of calcidiol to the active form of vitamin D (calcitriol) have been found throughout the central nervous system [39].

The finding of the considerably high prevalence of vitamin D deficiency (40 %) and insufficiency (39 %) in Iranian students is in line with the work of other groups in Iran demonstrating 78 % vitamin D deficiency (serum 25(OH)D < 20 ng/mL) in children and adolescents aged 8–18 years from Tehran [40], and 91.7 % in similar population during autumn and winter [41]. The results necessitate interventions for vitamin D supplementation or vitamin D fortifications in Iran.

The main limitation of this study is its cross-sectional design which does not demonstrate the causality of association between psychiatric distress and vitamin D deficiency. A reverse causation could be even assumed; meaning that psychiatric distress resulted in less outdoor activity and hence reduced vitamin 25(OH)D concentrations. However, outdoor physical activity and sedentary behaviors such as watching TV, working with computer, and sleeping duration were similar in vitamin D deficient and vitamin D sufficient children. Strengths of our study include its novelty in the pediatric age group, the large sample size and generalizability.

At present time, it is premature to conclude that vitamin D deficiency is related to occurrence of psychiatric distress in children. Until results of prospective studies confirm the causality, it is hard to recommend vitamin D supplementation in adolescents with mental problems. However, as low levels of 25(OH) D have been documented in several studies in Iran, prevention and control of vitamin D deficiency could be suggested as a health priority. Future studies could determine if vitamin D supplementation might reduce psychiatric distress by increasing 25(OH) D levels.

## Appendix 1

### The questions and codes to categorize students for psychiatric distress and violence behaviors:

“During the past 6 months, how often did you experience angriness/anxiety/poor quality sleep/confusion so that you cannot do your daily activity?”(Response options were: almost every day, more than once a week, almost every week, and almost every month, rarely or never). (Almost every day, more than once a week, almost every week [yes]; almost every month, rarely or never [no]). Sadness/Depression: “During the past 12 months, did you ever feel so sad or hopeless almost every day for 2 weeks or more in a row that you stopped doing your usual activities?” (Response options were: yes, no). Worry: “During the past 12 months, how often have you been so worried about something that you could not sleep at night?” (Response options were: never, rarely, sometimes, most of the time, and always) (never, rarely, sometimes [no]; most of the time, and always [yes]).

Violence behavior section included physical fight, bully, or getting bullied as below: “During the past 12 months, how many times you had physical fight?” (Response options were: none, 1 time, 2 times, 3 times, 4 times) (none[No], 1 or 2 or 3 or 4 times [Yes]). “During the past 3 months, how many times you were bullied, or got bullied?”(Response options were: none, 1–2 times, 2–3 times, 4 times or more).(none [No]; 1–2 times, 2–3 times, 4 times or more[Yes])

Self- perceived general health status was assessed as below: “In general, would you say your health is:” (Response options were: excellent, good, fair, poor) (excellent or good [good status]; fair or poor [bad status]).
